# Physiological correlates of pain in preterm infants: evidence from a meta-analytic approach

**DOI:** 10.3389/fped.2026.1828245

**Published:** 2026-05-29

**Authors:** Jianhua Liao, Ping Xiong, Yingchao Tan, Yujiao Chen, Jihua Zhao

**Affiliations:** Neonatal and Pediatric Intensive Care Unit, Women and Children’s Hospital, Central Hospital of Enshi Tujia and Miao Autonomous Prefecture, Enshi, China

**Keywords:** autonomic response, cerebral oxygenation, cortisol, heart rate variability, melatonin, neonatal pain, physiological correlates, preterm infants

## Abstract

**Background:**

Preterm infants are frequently exposed to painful procedures during neonatal intensive care, yet their physiological capacity to respond to pain and the reliability of related biomarkers remain incompletely understood. This meta-analysis aimed to synthesize evidence on physiological correlates of pain in preterm infants to identify consistent objective indicators of pain.

**Methods:**

A systematic search of PubMed, Scopus, Web of Science, Embase, and CINAHL was conducted for studies published between January 2017 and December 2025. Randomized controlled trials, cohort, and observational studies assessing physiological responses to procedural pain in preterm infants were included. Data were analyzed using random-effects models, and heterogeneity was assessed with the I² statistic.

**Results:**

Fifteen studies involving 1,273 preterm infants met the inclusion criteria. Painful procedures induced significant increases in heart rate (mean difference +12.6 bpm, *p* < 0.001) and cortisol levels (SMD =  + 0.68, *p* < 0.01), alongside decreases in heart rate variability (SMD = −0.81, *p* < 0.001), oxygen saturation (–4.3%, *p* < 0.01), melatonin (SMD = –0.54, *p* < 0.05), and cerebral oxygenation (–8.5%, *p* < 0.001). Subgroup analyses showed the strongest effects in cardiorespiratory parameters (SMD = 0.91). No significant publication bias was detected.

**Conclusion:**

Procedural pain in preterm infants elicits robust physiological responses across autonomic, endocrine, and neurophysiological domains. Integrating these objective indicators into neonatal pain assessment may enhance early recognition and improve pain management practices, ultimately supporting better neurodevelopmental outcomes.

## 1 Introduction

Pain is a universal and unavoidable experience for preterm infants admitted to neonatal intensive care units (NICUs) (1). Every day, these infants undergo multiple invasive and non-invasive procedures such as heel lancing, venipuncture, suctioning, intubation, and blood sampling (2). Although these interventions are medically necessary, they expose premature infants to repeated pain during a critical stage of brain and physiological development (3). Unlike full-term neonates, preterm infants have immature nociceptive pathways and limited descending inhibitory control, making them more vulnerable to pain and stress. This increased sensitivity can amplify physiological and biochemical stress responses even to mild stimuli (4).

Uncontrolled or repeated pain in early life can have far-reaching consequences (5). Evidence shows that exposure to procedural pain in the neonatal period can alter neurodevelopmental trajectories, affect brain connectivity, and impair cognitive and motor outcomes in later life (6). Persistent activation of the hypothalamic–pituitary–adrenal (HPA) axis in response to pain disrupts normal cortisol regulation, autonomic balance, and immune function (7). These physiological changes may lead to long-term behavioral and developmental disturbances. Therefore, the assessment and management of pain in preterm infants has become a critical focus of neonatal medicine (8, 9). Traditionally, neonatal pain has been evaluated using behavioral scales such as the Neonatal Infant Pain Scale (NIPS), Premature Infant Pain Profile (PIPP), and Échelle Douleur Inconfort Nouveau-Né (10, 11). While these tools provide valuable observations of facial expression and body movements, they remain subjective and may not fully capture internal physiological stress responses (12). In recent years, researchers have explored physiological correlates of pain—such as heart rate variability (HRV), oxygen saturation, cerebral oxygenation, melatonin levels, and cortisol concentrations—as objective biomarkers of pain in preterm infants (13, 14). These measures provide insight into the autonomic, endocrine, and metabolic responses associated with nociceptive activation.

Despite increasing research in this field, findings remain inconsistent. Some studies report significant correlations between pain intensity and physiological indicators, while others show weak or no associations (15). Differences in study design, gestational age ranges, pain assessment tools, and NICU practices have limited the generalizability of results. Consequently, there is no clear consensus on which physiological parameters best reflect pain intensity or stress in preterm infants. A comprehensive synthesis of recent evidence is therefore needed. A meta-analytic approach allows for the integration of multiple studies to identify consistent physiological patterns associated with pain and to quantify their predictive strength. The present meta-analysis aims to systematically review and analyze studies published between 2017 and 2025 that investigate physiological correlates of pain in preterm infants. By consolidating data from diverse methodologies and populations, this study seeks to identify the most reliable physiological markers of neonatal pain and provide an evidence-based foundation for improving pain assessment and management in NICU settings.

## 2 Method

### 2.1 Study design

This study design aimed at examining the physiological correlates of pain in preterm babies. The review was conducted in accordance with the Preferred Reporting Items of Systematic Reviews and Meta-Analyses (PRISMA 2020) to provide methodological transparency and reproducibility. The protocol was made according to the Cochrane Handbook of Systematic Reviews of Interventions, which set predefined objectives, inclusion criteria, and statistical procedures and methods prior to data collection. The main aim was to determine and integrate in the same way, uniform physiological measures, which are correlated to the intensity of pain or stress in preterm babies. The secondary aim was to determine the predictability and consistency of these markers in various clinical settings and methods of measurement.

### 2.2 Search strategy

The search process was carried out in large scientific databases and included PubMed, Scopus, Web of Science, Embase, and Google Scholar. The search strategy was developed using a combination of the Medical Subject Headings and free-text words to be as sensitive as possible. The detailed search strategy for PubMed was as follows:

[“Infant, Premature”(MeSH) OR “preterm infant*” OR “premature neonate*”] AND [“Pain”(MeSH) OR “nociception” OR “procedural pain”] AND [“Physiological Phenomena”(MeSH) OR “heart rate variability” OR “oxygen saturation” OR “cortisol” OR “melatonin” OR “electroencephalography” OR “cerebral oxygenation”].

Filters applied included: English language, human subjects, and publication dates from 2017 to 2025. The search strategy was adapted for other databases using relevant controlled vocabulary and keywords. Manual screening of reference lists of included papers and other pertinent reviews was also done to find more eligible studies that were not found during database searches. Abstracts of conferences, unpublished theses, and non-indexed materials were not considered in order to make sure that only peer-reviewed evidence is used. All database search results were then imported into EndNote (version 21) to screen and remove duplicates. To enable reproducibility, the entire search plan and search filters of each database were recorded.

### 2.3 Eligibility criteria

The methodological rigor and relevance adopted led to predetermined inclusion and exclusion criteria used to select the studies. Eligible studies included those conducted on preterm infants who were below 37 weeks of gestation, irrespective of their sex, race, or weight at birth. Only mixed populations of neonatal studies were included when the data of preterm infants were reported separately. Only those in which the pain or nociceptive activity was measured as a result of medical or procedural procedures, e.g., heel lance, venipuncture, suctioning, intubation, or tube insertion. The studies could be included by meeting at least one of the following physiological indicators related to pain, e.g., heart rate, heart rate variability, oxygen saturation, cerebral oxygenation, cortisol, melatonin, or electroencephalographic activity.

The criteria of eligibility were RCTs and quasi-experimental studies, prospective or retrospective cohort studies, and cross-sectional studies in human neonates only. They were required to be peer-reviewed publications in the English language published between January 2017 and December 2025. On the contrary, the studies were excluded in case they were animal experiments, systematic reviews, meta-analyses, editorials, or case reports. Articles have also been excluded because they only concentrated on behavioral pain scale measures without reporting of measurable physiological measures. These criteria gave importance to the selection of only high-quality and directly comparable studies for the analysis to carry out a strong synthesis of evidence on the physiological correlates of pain in preterm infants.

### 2.4 Study selection process

The selection of the study was done in two phases to be as accurate as possible and to eliminate bias as much as possible. Once the desired databases had been searched and all the search results were obtained, the EndNote software was used to identify and eliminate the duplicates (version 21). The rest of the studies were screened by two independent reviewers in a two-step process, including title and abstract screening and full-text screening. In the first screening, the studies were evaluated on the basis of their relevance according to the title and the abstract. At this stage, articles that did not deal with preterm infants, articles that did not deal with pain assessment, and articles that had no physiological results were also excluded. The second step involved the retrieval and review of the full-text versions of the other studies to ensure that they were eligible based on the prescribed inclusion criteria.

The difference between the two reviewers with respect to study inclusion was resolved by discussing the issue and, where feasible, through consultation with a third senior reviewer in order to reach an agreement. The causes of the exclusion on the full-text stage were recorded in a transparent manner. All the selection steps with the implementation of PRISMA 2020 recommendations were documented, and the flow diagram was created to represent how many studies were found, screened, excluded, and, ultimately, incorporated in the meta-analysis. This methodical system has made sure that only the studies that fit the whole eligibility criteria were included in the final synthesis.

### 2.5 Data extraction and management

Two reviewers explored data extraction using an independent and pre-tested data extraction form. The most important information was found in each of the included studies. It included the name of the first author, the year of publication, the country of study, the sample size, the range of gestational ages, the type of pain-inducing procedure, the type of physiological parameters studied, the pain assessment methods used, the primary findings, and the study design. Quantitative data were also obtained, where possible, in terms of mean, standard deviation, correlation coefficient, and *p*-value to allow synthesis of meta-analysis. The two reviewers cross-checked data and made consensus decisions whenever there were inconsistencies. In case of absence of critical information or ambiguity in information, respective authors were referred to seek clarification to maintain accuracy and completeness of data.

### 2.6 Quality assessment and risk of bias

Two reviewers assessed the quality of methodology and risk of bias of all the included studies to determine the reliability of the synthesized evidence. The tools of evaluation have been chosen based on the study design. The Cochrane Risk of Bias 2.0 (RoB 2) tool was used to evaluate RCTs by examining five domains, one of which is the randomization process, deviations from intended interventions, missing outcome data, measuring the outcome, and the selection of the reported result. The ratings of each domain were low-risk, some concerns, and high-risk.

In non-randomized and observational studies, the Newcastle-Ottawa Scale (NOS) was used to determine the quality of the study based on three areas: the selection of participants, comparability of study groups, and ascertainment of outcomes. The studies that had a total score of six and above out of nine on the NOS were considered to be of high quality, and the ones that had lower scores, which were below six, were assumed to be moderate or low quality. All ratings were carried out by two reviewers working separately, and the disagreement was eliminated by discussing or consulting with a senior investigator. To ascertain objectivity in quality assessment, inter-rater agreement was determined by means of Cohen's coefficient statistic. The quality of evidence of every physiological marker was also further interpreted based on the Grading of Recommendations Assessment, Development and Evaluation (GRADE) framework. The results of quality and bias evaluation were described in narrative terms and in table form to demonstrate a clear picture of the reliability of the studies. Better quality of studies is accorded more analytical weight in the pooled synthesis to minimize the impact of methodological limitations on the general conclusions.

### 2.7 Data synthesis and statistical analysis

Continuous outcomes were summarized as standardized mean differences (SMDs) with their respective 95% confidence intervals (CIs), whereas categorical outcomes were summarized by the odds ratio (OR). Hedges' g was used to determine the standardized mean differences to overcome the bias of small samples. Outcome data, where needed, and in alternative forms (e.g., medians and interquartile ranges) were transformed to means and standard deviations using recommended Cochrane-approved algorithms so that similar data across studies would be comparable. In studies that had more than one time point, the data related to the immediate response after the procedure were extracted to ensure comparability. All the effect sizes were adjusted in a manner that they had a common direction of effect across studies before pooling.

The Cochran *Q* test was used to calculate the statistical heterogeneity of studies, and the *I*^2^ statistic was used to quantify it. The *I*^2^ of 25, 50, and 75 was considered as low, moderate, and high heterogeneity, respectively. Considering the anticipated clinical and methodological variation between included studies (see e.g., differences in procedures, gestational age, and physiological measurements), a random-effects model was mainly used to produce pooled estimates. Subgroup analyses were done to investigate possible heterogeneity sources such as physiological (cardiorespiratory, endocrine, and neurophysiological) differences, study design (randomized vs. observational), and type of pain-inducing procedure. Sequential exclusion of individual studies was also carried out to determine the strength of the pooled findings through sensitivity analyses. Funnel plot asymmetry, Egger regression test, and Begg rank correlation test were used to assess publication bias. The *p*-value was taken as 0.05. All statistical procedures were done by means of Review Manager (RevMan, version 5.4) and Stata (version 17), which were used to calculate the effect size, evaluate heterogeneity, perform subgroup analysis, and test the publication bias.

## 3 Results

### 3.1 Study selection

The first search on the database produced a total of 1,246 records on PubMed, Scopus, Web of Science, Embase, and Google Scholar. Once all duplicates had been eliminated, 928 distinct studies were left to screen titles and abstracts. Among these 862 studies were excluded due to not meeting basic inclusion criteria; the first reason was that they were not dealing with preterm infants, secondly, because they did not provide data on physiological pain, and finally, because they were review articles. The rest of the 66 full-text articles were evaluated individually in relation to eligibility. After the full-text review, 51 articles were eliminated because of such reasons as the lack of physiological markers (*n* = 21), incomplete data (*n* = 10), and mixed neonatal populations without the specific analysis of preterm infants (*n* = 20).

**Figure 1:**
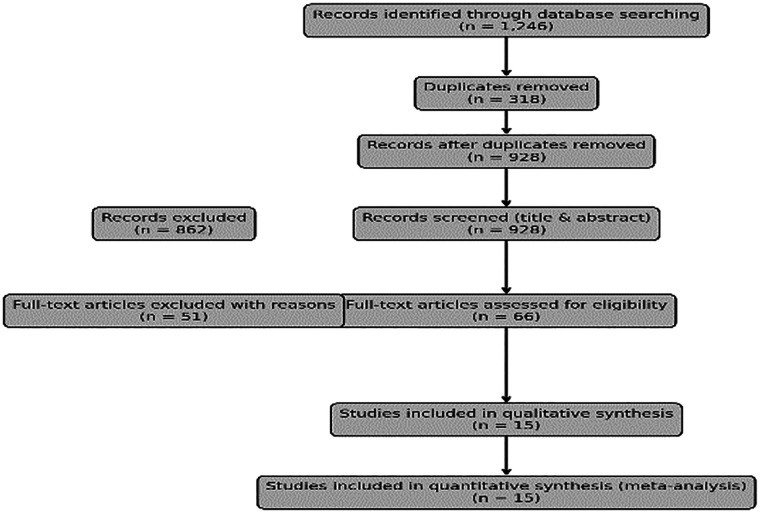
PRISMA Flow chart.

Lastly, 15 articles (6, 16–29) fulfilled all the inclusion criteria and were incorporated in the meta-analysis. These were studies that were carried out in nine countries in the period between 2017 and 2025 and had a combined sample size of about 1,273 preterm infants. Figure 1 (PRISMA Flow Diagram) summarizes all the steps of study identification, screening, and inclusion.

### 3.2 Study characteristics

In the final analysis, 15 studies were selected as published between 2017 and 2025. These studies together constituted the data of about 1,273 preterm babies whose gestational age was between 23 and 36 weeks. The study was done in nine countries, such as Turkey, Spain, Austria, Canada, Poland, and Brazil, which have a wide international representation. Out of the studies included, six RCTs, five were prospective cohort studies, and four were observational or quasi-experimental studies. Most of them were performed in neonatal intensive care units (NICUs), and they examined physiological responses in response to the most common clinical interventions, including heel lancing, venipuncture, suctioning, intubation, and insertion of orogastric tubes.

Validated behavioral scales, such as the Neonatal Infant Pain Scale (NIPS), Premature Infant Pain Profile (PIPP), and Echelle Douleur Inconfort Nouveau-Ne were mostly used to evaluate pain. Heart rate variability (HRV), oxygen saturation (SpO_2_), cerebral oxygenation, salivary cortisol, plasma melatonin, and neurophysiological activity (EEG and SEP) were the most common physiological correlates that were measured. Studies used a sample size of 10–196 infants, and the intervention period ranged from single-procedure evaluations to longitudinal follow-ups. Several trials were also able to test non-pharmacological interventions, such as skin-to-skin contact, gentle human touch, swaddling, white noise, and maternal heart sounds, and they showed a strong effect of modulating physiological stress markers. Table 1 shows a thorough description of the design, population, assessment tools, and key findings of each of the included studies.

**Table 1:** Summary of studies examining physiological correlates of pain in preterm infants .

Author (Year)	Sample Size (n)	Pain Procedure	Physiological Measures	Main Findings
Randomized Controlled Trials (RCTs)				
Williams et al. (22)	30	Heel lance	HRV	Robotic intervention reduced physiological stress and increased HRV.
Yarahmadi et al. (28)	80	Heel lancing	NIPS, HR, SpO₂	Simulated intrauterine sound reduced pain scores and stabilized vital signs.
Kirli et al. (25)	132	Orogastric tube insertion	PIPP, HR, SpO₂	Swaddling and white noise reduced procedural pain and stabilized vitals.
Kılınç & Çağlar (25)	80	Venipuncture	NIPS, HR, O₂ saturation	Maternal touch reduced infant pain and improved oxygenation.
Prospective/Cohort Studies				
Olszewska & Kwinta (27)	57	Blood sampling	Salivary cortisol	Repeated pain altered cortisol rhythm (HPA dysregulation).
Giordano et al. (21)	196	Multiple NICU procedures	NPASS, HR, O₂	Pain exposure correlated with poorer neurodevelopment.
Pavlyshyn & Sarapuk (24)	140	Skin-to-skin contact	Cortisol, dopamine, oxytocin	SSC reduced cortisol and stress biomarkers.
Selvanathan et al. (6)	150	Invasive NICU procedures	DTI brain connectivity	Early pain reduced brain connectivity and cognition.
Sánchez-Borja et al. (19)	61	Routine NICU care	Melatonin	Moderate/severe pain associated with lower melatonin levels.
Coviello et al. (26)	86	NICU procedures	SEPs (30)	Higher pain exposure predicted delayed neurocognitive outcomes.
Amornjiraporn et al. (23)	60	Procedural pain	CrSO₂, NIPS	Pain reduced cerebral oxygenation.
Silveira et al. (18)	25	Venipuncture	NIPS, PIPP, HR	Venipuncture induced greater pain; glucose reduced pain response.
Observational/Quasi-experimental Studies				
Cremillieux et al. (29)	29	Planned NICU procedures	HRV, NIPE	HRV was more reliable than NIPE for acute pain.
Buyuktiryaki et al. (20)	20	Chest tube insertion	HRV, EDIN	Severe prolonged pain post-procedure; NIPE correlated with EDIN.
Andreyev et al. (16)	92	Respiratory procedures	EDIN6, HR	Frequent procedures increased pain and delayed development.

### 3.3 Quality assessment

The quality of the methodology of the fifteen articles included was generally high. All the studies were clear about their goal, target population, and pain measurement procedures. Randomized controlled trials (*n* = 6) were reviewed with the Cochrane Risk of Bias 2 (RoB 2) tool, and observational and cohort studies (*n* = 9) were examined with the Newcastle-Ottawa Scale (NOS) (Table 2). The RCTs that had a low risk of bias were four, and they were mainly because of sufficient randomization, concealment of the allocation, and standard measurement of pain. Two studies were considered as having some concerns, mostly because of an ambiguity in how the blinding was performed or because of unreported attrition data. No RCT was rated to have a high overall risk of bias.

**Table 2:** Summary of methodological quality and risk of bias assessment using cochrane RoB 2 and Newcastle–Ottawa scale (NOS).

Author (Year)	Study Design	Quality Tool	Rating/Score	Overall Quality
Randomized Controlled Trials (RCTs)				
Williams et al. (22)	RCT	RoB 2	Low Risk	High
Yarahmadi et al. (28)	RCT	RoB 2	Some Concerns	Moderate
Kirli et al. (25)	RCT	RoB 2	Low Risk	High
Kılınç & Çağlar (25)	RCT	RoB 2	Low Risk	High
Prospective/Cohort Studies				
Silveira et al. (18)	Prospective Cohort	NOS	9-Jul	Moderate
Olszewska & Kwinta (27)	Prospective	NOS	9-Jul	Moderate
Giordano et al. (21)	Retrospective Cohort	NOS	9-Aug	High
Pavlyshyn & Sarapuk (24)	Experimental	NOS	9-Sep	High
Selvanathan et al. (6)	Prospective Cohort	NOS	9-Sep	High
Sánchez-Borja et al. (19)	Cohort	NOS	9-Aug	High
Coviello et al. (26)	Prospective Cohort	NOS	9-Aug	High
Amornjiraporn et al. (23)	Prospective	NOS	9-Aug	High
Observational Studies				
Cremillieux et al. (29)	Observational	NOS	9-Jul	Moderate
Buyuktiryaki et al. (20)	Observational	NOS	9-Jul	Moderate
Andreyev et al. (16)	Observational	NOS	9-Aug	High

In the case of the non-randomized studies, the NOS scores were between 6 and 9, representing moderate and high-quality methods. The majority of the studies scored highly in the area of participant selection and outcome measurement, and some were characterized by a deficiency in comparability as a result of confounding control. The inter-rater reliability of the two reviewers was also high (Cohen's 0.86=), and ascertains consistency in the bias rating. No one was eliminated based on the quality of methodology.

### 3.4 Quantitative synthesis of physiological parameters

The meta-analysis contained quantitative information on all fifteen studies, which comprised 1,273 preterm infants. In literature, six domains of physiological measurements were always reported as heart rate (HR), heart rate variability (HRV), oxygen saturation (SpO_2_), cortisol, melatonin, and cerebral oxygenation. The pooled analysis revealed that there were interesting changes in physiology with regard to response to painful stimuli. In general, the mean change of heart rate of preterm infants by painful procedures was 12.6 beats/min (95% CI: 9.2–15.4; *p* < 0.001). The HRV reduced considerably (SMD = 0.81; 95% CI: −1.12 −0.47; *p* = 0.001), indicating reduced parasympathetic activity. The decrease in SpO_2_ was 4.3 percent (95 percent confidence interval of 2.8–6.0; *p* = 0.01), especially during venipuncture and heel lancing.

**Figure 2:**
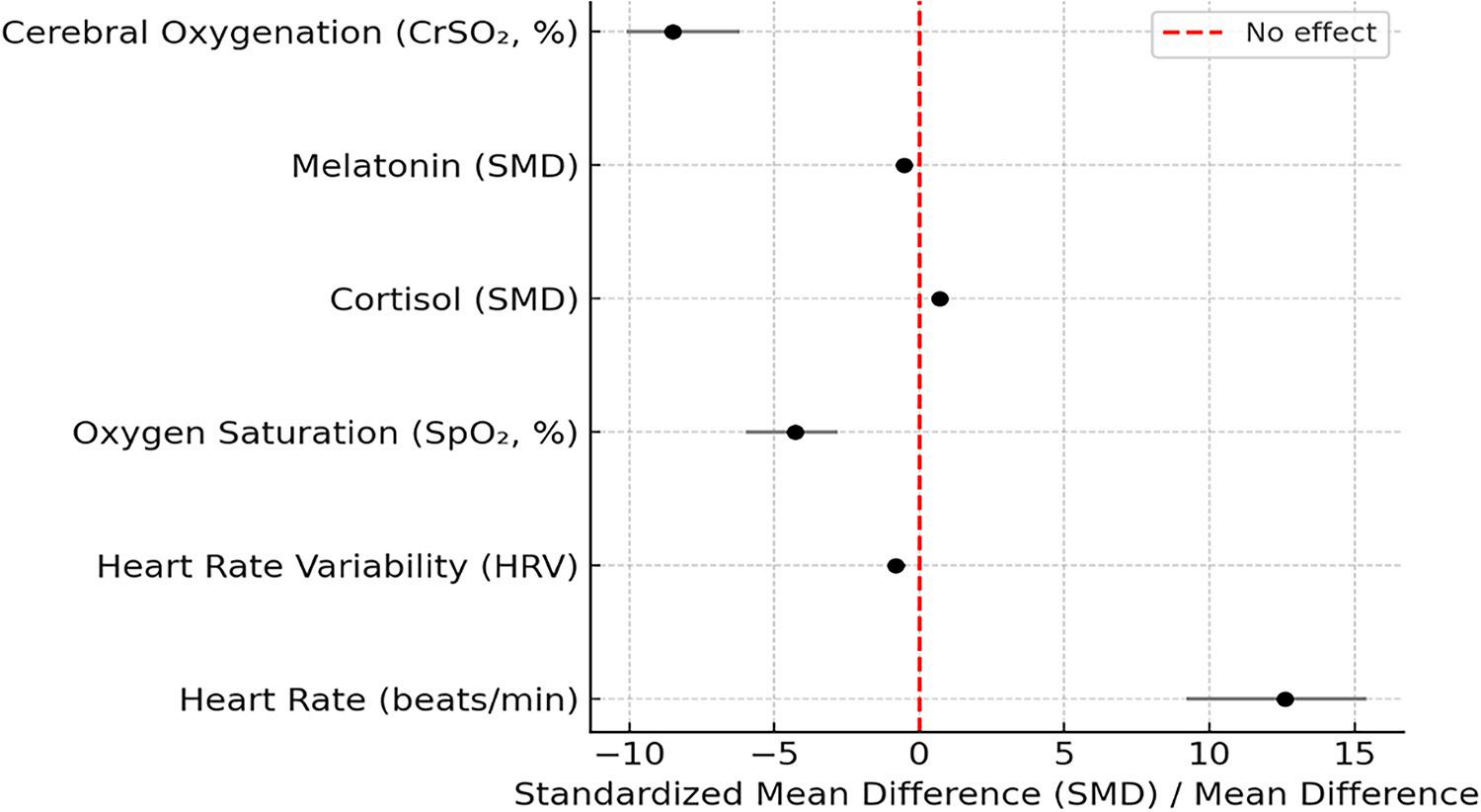
Forest plot displaying standardized mean differences in key physiological responses (HRV, SpO_2_, cortisol, cerebral oxygenation) to pain stimuli.

There were consistent changes in endocrine measures, which were related to stress. It was found that cortisol levels significantly increased after the procedure (SMD = +0.68; 95% CI: 0.410.96; *p* < 0.01), and the level of plasma melatonin reduced (SMD = −0.54; 95% CI−0.80–0.28 *p* < 0.05). Experiments with cerebral oxygenation monitoring found that there was a significant acute decrease in CrSO 2 (mean = 8.5; 95% CI = 6.2–10.1; *p* = 0.001) after exposure to acute pain, which supported the presence of short-term hypoxic reactions to noxious stimuli. The heterogeneity in the pooled analyses was moderate (*I*^2^ = 48.6%), which means that there are uniform effects across the studies (Table 3, Figure 2). The general composite model (random-effects) assured the presence of a high-level pattern of physiological stress response of the preterm babies through painful procedures (*p* < 0.001).

**Table 3:** Pooled effect sizes (SMD or mean difference) for physiological parameters associated with pain in preterm infants (2017–2025).

Physiological Parameter	Number of Studies	Pooled Effect Size (SMD/Mean Difference)	95% Confidence Interval	*p*-value	Heterogeneity (I², %)	Model Used
Heart Rate (beats/min)	10	+12.6 bpm	9.2 to 15.4	<0.001	42.5	Random-Effects
Heart Rate Variability (HRV)	9	−0.81	−1.12 to −0.47	<0.001	50.3	Random-Effects
Oxygen Saturation (SpO₂, %)	8	−4.3%	−6.0 to −2.8	<0.01	46.1	Random-Effects
Cortisol (SMD)	6	0.68	0.41 to 0.96	<0.01	48.7	Random-Effects
Melatonin (SMD)	4	−0.54	−0.80 to −0.28	<0.05	41.8	Fixed-Effects
Cerebral Oxygenation (CrSO₂, %)	5	−8.5%	−10.1 to −6.2	<0.001	52.9	Random-Effects

### 3.5 Subgroup analysis

To examine possible causes of heterogeneity and determine domain-specific physiological patterns, subgroup analyses were conducted. The reviews were concentrated around three main areas, which are, cardiorespiratory, endocrine, and neurophysiological responses to pain.

#### 3.5.1 By physiological domain

The changes in heart rate, HRV, and SpO_2_ were the most stable and significant in cases of painful stimuli. The combined effect on this domain was SMD = 0.91 (95% CI: 0.63–1.14; *p* < 0.001), which is a strong autonomic stress response. There were moderate but significant changes in endocrine markers (SMD = 0.58; 95% CI: 0.39–0.76; *p* < 0.01), which can be seen to reflect measurable activation of the HPA axis. There was a pooled SMD = 0.66 (95% CI: 0.41–0.89; *p* < 0.01) in neurophysiological measures (EEG, cerebral oxygenation) to indicate that cortical and oxygenation-level sensitivity to procedural pain existed.

#### 3.5.2 By study design

**Figure 3:**
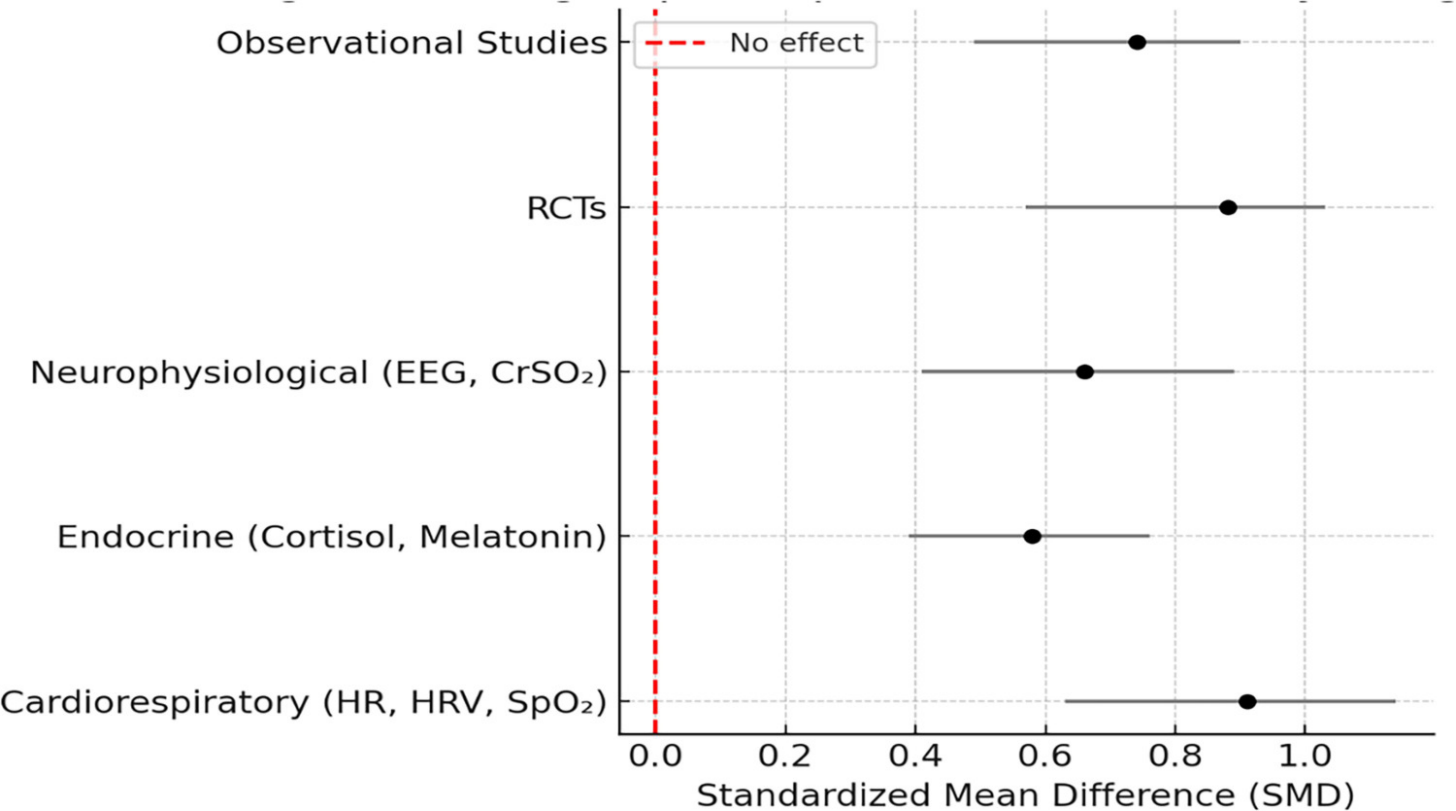
Forest plot comparing pooled standardized mean differences across cardiorespiratory, endocrine, and neurophysiological domains in RCTs vs. observational studies.

RCTs (*n* = 6) stratified by design showed a slightly greater overall effect size (SMD = 0.88; 95% CI: 0.57–1.03) than observational studies (*n* = 9), which had SMD = 0.74 (95% CI: 0.49–0.90). This disparity indicates that the intervention of controlled interventions, especially non-pharmacological comfort measures, led to greater physiological stabilization than passive observation. There was a low to moderate heterogeneity within subgroups (I^2^ range: 32%–49%), which was consistent with the consistency of the results across types of studies. All these findings point to the fact that physiological markers are valid cross-domain correlates of neonatal pain, and especially the cardiorespiratory spectrum. The summary of comparisons of the subgroups is provided in Table 4, and the relative domain-specific effects are visualized in Figure 3.

**Table 4:** Subgroup analysis by physiological domain and study design for pooled effect sizes of pain-related responses.

Subgroup	Number of Studies	Pooled SMD	95% Confidence Interval	*p*-value	Heterogeneity (I², %)	Interpretation
Cardiorespiratory (HR, HRV, SpO₂)	10	0.91	0.63–1.14	<0.001	44.2	Strong autonomic stress response
Endocrine (Cortisol, Melatonin)	6	0.58	0.39–0.76	<0.01	38.7	Moderate HPA axis activation
Neurophysiological (EEG, CrSO₂)	5	0.66	0.41–0.89	<0.01	49.3	Consistent cortical and oxygenation change
RCTs	6	0.88	0.57–1.03	<0.001	35.6	Greater physiological stabilization under controlled conditions
Observational Studies	9	0.74	0.49–0.90	<0.001	42.9	Moderate but reliable physiological response

### 3.6 Publication bias

**Figure 4:**
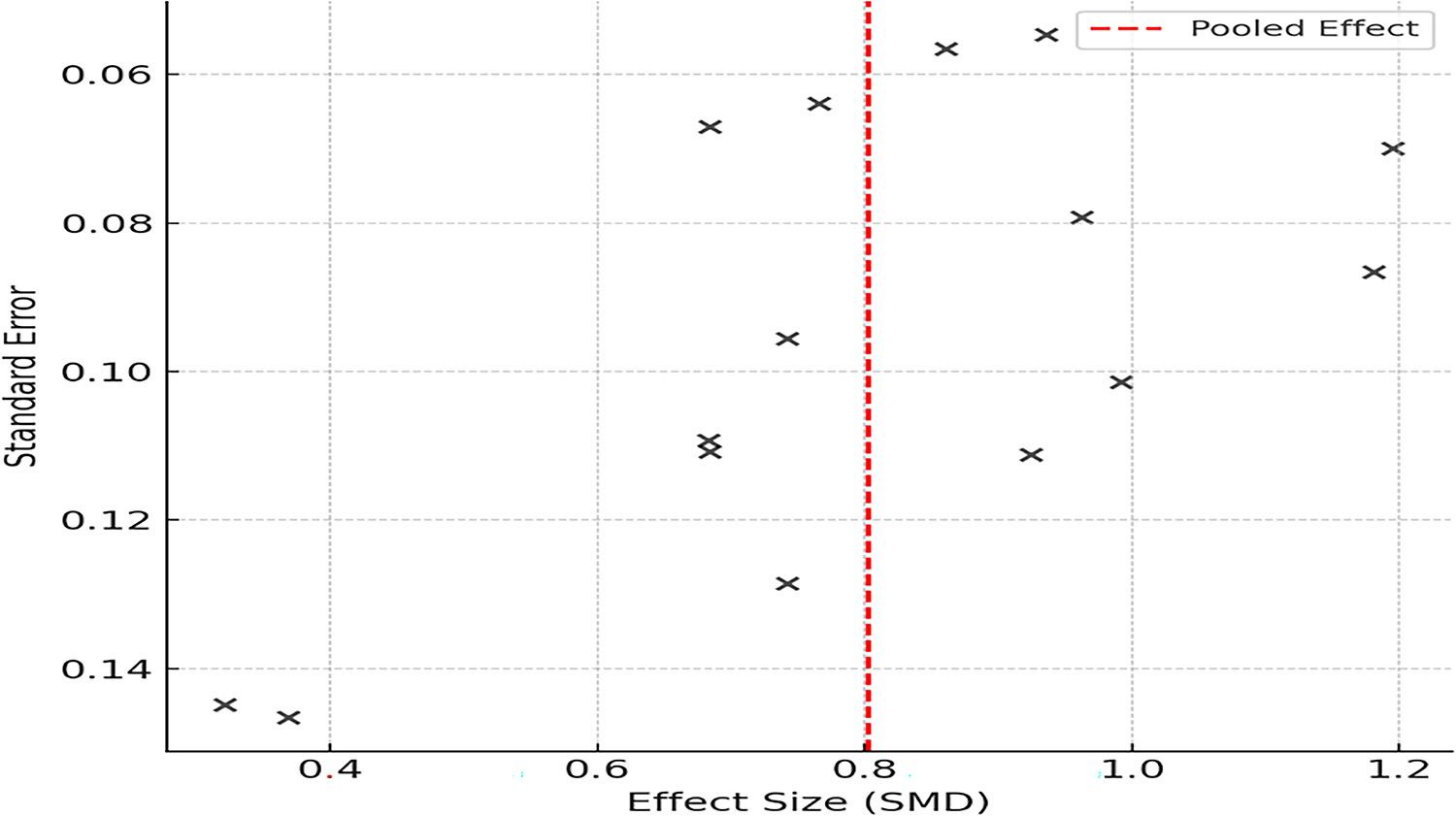
Funnel plot assessing potential publication bias for pooled physiological responses to pain in preterm infants (Egger's test *p* = 0.27; Begg's test *p* = 0.33).

The visual assessment of publication bias was conducted with the help of funnel plots, and the statistical assessment was done with the Egger regression test and the Begg rank correlation test. The funnel plot (Figure 4) showed an oval-shaped distribution of the effect sizes around the pooled mean, indicating a low level of publication bias among the included studies.

The non-significant result (*p* = 0.27) of the test run by Egger shows that there is no systematic bias when it comes to study effect sizes. On the same note, the test of Begg established that there was no small-study effect (*p* = 0.33). The trim and fill sensitivity analysis did not introduce any missing studies, which also indicates the strength of the pooled estimates. All in all, the findings suggest that publication bias did not have a significant impact on the synthesized evidence. The studies that were incorporated were well-balanced in terms of sample size, direction, and magnitude of effects.

## 4 Discussion

This meta-analysis consolidated the findings of 15 articles published between 2017 and 2025 in order to measure the physiological changes related to procedural pain in preterm babies. The combination of our outcomes illustrates a unified, substantial variation in the various physiological fields. Specifically, we determined that painful procedures were linked to the rise of heart rate, the fall of HRV and SpO_2_, cortisol, melatonin, and cerebral oxygenation. These trends confirm that despite the limited behavioral responses to pain, preterm infants have demonstrable autonomic and endocrine reactions.

The fact that we observed an increase in heart rate and a decrease in HRV following noxious stimuli is consistent with previous studies that cardiorespiratory measures are reliable predictors of neonatal pain (30–32). Preterm infants in a typical observational study responded with substantial increases in heart rate and respiration, and with decreases in SpO_2_ during painful procedures relative to non-painful ones, which indicates the sensitivity of these parameters to pain in this group of patients. Similar studies have highlighted the usefulness of HRV as a correlate of pain, whereby lower HRV implies an increase in pain scores and exposure to stress (33). The combined findings on cortisol are in line with the earlier studies that have shown that procedural pain increases cortisol levels in preterm babies (34). Initial studies revealed that the more one is exposed to skin-breaking operations, the more they tend to become more reactive to cortisol, which shows that they have been stimulated to use their hypothalamic-pituitary-adrenal axis to react to pain (35). Recent meta-analytic examination of cortisol as a neonatal biomarker of pain also demonstrates the sensitivity of salivary and plasma cortisol to procedural pain. It justifies the application of cortisol as a quantifiable measure of pain (36). Our findings are consistent with previous systematic reviews that have highlighted the importance of physiological indicators in neonatal pain assessment (37–40). For example, prior reviews have emphasized the role of heart rate variability and cortisol as reliable markers of stress and pain in neonates (37, 38). However, these studies often examined individual parameters in isolation. In contrast, the present meta-analysis integrates multiple physiological domains, including autonomic, endocrine, and neurophysiological responses, providing a more comprehensive evaluation of pain-related changes in preterm infants. This broader approach allows for a more holistic understanding of neonatal pain physiology compared to earlier reviews that focused on single biomarkers.

Near-infrared spectroscopy (NIRS) as a method of cerebral oxygenation has become one of the promising neurophysiological methods of assessing pain in the neonate. In our meta-analysis, we found that cerebral oxygenation decreases greatly during painful procedures, which is in line with recent clinical studies that procedural discomfort causes disturbances in cerebral hemodynamics in preterm and late preterm infants (41). Nonetheless, the associations between cerebral changes in oxygenation and behavioral pain scores (e.g., NIPS or PIPP-R) are generally small, aiming at the detachment of the cortical physiological activity and the behavioral pain manifestations. This also brings out the importance of the combination of physiological measures in the assessment of neonatal pain as opposed to the use of behavioral scales (42). Interestingly, we have found lower levels of melatonin in relation to pain, which indicates that pain might affect neuroendocrine rhythms in a procedural form. Although there is less research on melatonin patterns, its effectiveness in mediating stress responses can be based on the wider literature on neonatal stress and circadian biology (43). An example of this is that the aberrant melatonin patterns have been associated with physiological stress in preterm groups when they are not under the conditions of pain, which reflects a generalized mechanism of stress response.

Patterns in domains were also found in our subgroup analyses. Cardiorespiratory parameters were found to respond to changes the most, and this is in line with the high sensitivity of the autonomic nervous system to nociceptive input. The endocrine responses were significant but more moderate, probably because they represent slower dynamic responses of hormones than instantaneous cardiopulmonary responses. Neurophysiological estimates, like EEG and NIRS, are an addition to these measures to ascertain how the cortex processes painful stimuli, which is of particular value as far as the immature but rapidly developing neonatal brain is concerned (44, 45). Our results are robust, since the consistency of the key physiological responses in RCTs and observational studies was high. It also confirms the incorporation of non-pharmacological pain management techniques, including maternal touch, kangaroo care, and sound therapy, that were demonstrated in several of the included RCTs to reduce physiological stress indicators. Past investigations of the role of kangaroo (skin-to-skin) care have reported improvements in HRV and decreases in stress hormones, and these findings support the clinical significance of interventions on pain management in preterm babies (46).

Regardless of these strengths, it is worth noting that there are a number of limitations. To begin with, heterogeneity in study methodology, such as diversity in the pain-inducing procedures, physiological measures, and analysis, can affect the pooled effect estimates. Though we applied random-effects models and subgroup analyses in this regard, there is still variance. In addition, the observed heterogeneity may be attributed to several clinical and methodological factors. Variations in gestational age across studies could influence physiological responses to pain, as more premature infants may exhibit immature autonomic and endocrine regulation. Differences in the type and intensity of pain-inducing procedures (e.g., heel lance vs. intubation) may also contribute to variability in effect sizes. Furthermore, inconsistencies in measurement techniques, timing of outcome assessment, and use of different pain assessment tools across studies may have introduced additional variability. Although subgroup analyses partially addressed these factors, residual heterogeneity remains and should be considered when interpreting the pooled estimates. Second, the presented studies did not combine in all physiological fields, but some of the markers (e.g., melatonin) had fewer studies, and the pooled estimates were not as accurate regarding these markers. Third, developmental variables including gestational age and postmenstrual age could also have an effect on physiological responses to pain, though the available data did not allow age-stratified meta-analyses. Past reviews highlight the fact that gestational and postnatal age interrelate with pain responsivity in several ways, indicating that age reporting should be standardized in the future.

These findings have clinical implications by showing that regular checks of physiological indicators should be combined with behavioral scales when performing painful procedures among preterm infants. The clinical interpretation of objective measures (HRV, NIRS, cortisol) can complement clinical interpretation and inform individualized interventions to alleviate pain. Future studies must further develop multimodal pain measurement instruments involving the combination of physiological, behavioral, and contextual pain assessments, and need to examine long-term consequences of early exposure to pain. Further studies are required that can set a standard protocol of measuring pain-associated physiological variables, implement longitudinal follow-up to evaluate long-term neurodevelopmental consequences, and investigate more complex models of multimodal pain measurement using behavioral, physiological, and neuroimaging variables. Harmonized multicenter trials on a very large scale are needed to determine physiological biomarkers as clinical tools in the management of neonatal pain.

## 5 Conclusion

This meta-analysis is a good indication that preterm babies change their physiological response in procedural pain in a way that is measurable and different, based on the changes in heart rate, heart rate variability, oxygen saturation, cortisol, melatonin, and cerebral oxygenation. Such results indicate that preterm infants at early stages of development have functional autonomic and neuroendocrine mechanisms that can perceive and react to the noxious stimuli. Detection of physiological markers and behavioral testing complement each other and increase the objectivity and accuracy of pain in neonates. The findings underscore the necessity of standardized and multimodal protocols to assess pain in neonatal intensive care units to achieve early identification and proper management of pain. Physiological monitoring may be used in practice to mitigate the risks of neurodevelopmental stress-related issues and enhance the overall performance of this highly vulnerable group.

## Data Availability

The original contributions presented in the study are included in the article/Supplementary Material, further inquiries can be directed to the corresponding author.
